# Impact of Paclitaxel-Eluting Balloons Compared to Second-Generation Drug-Eluting Stents for of In-Stent Restenosis in a Primarily Acute Coronary Syndrome Population

**DOI:** 10.5935/abc.20170142

**Published:** 2017-10

**Authors:** Guillaume Marquis-Gravel, Alexis Matteau, Brian J Potter, François Gobeil, Nicolas Noiseux, Louis-Mathieu Stevens, Samer Mansour

**Affiliations:** Centre Hospitalier de l'Université de Montréal

**Keywords:** Angioplasty, Balloon, Drug-Eluting Stents, Paclitaxel, Coronary Restenosis, Acute Coronary Syndrome

## Abstract

**Background:**

The place of drug-eluting balloons (DEB) in the treatment of in-stent restenosis (ISR) is not well-defined, particularly in a population of all-comers with acute coronary syndromes (ACS).

**Objective:**

Compare the clinical outcomes of DEB with second-generation drug-eluting stents (DES) for the treatment of ISR in a real-world population with a high proportion of ACS.

**Methods:**

A retrospective analysis of consecutive patients with ISR treated with a DEB compared to patients treated with a second-generation DES was performed. The primary endpoint was a composite of major adverse cardiovascular events (MACE: all-cause death, non-fatal myocardial infarction, and target lesion revascularization). Comparisons were performed using Cox proportional hazards multivariate adjustment and Kaplan-Meier analysis with log-rank.

**Results:**

The cohort included 91 patients treated with a DEB and 89 patients treated with a DES (74% ACS). Median follow-up was 26 months. MACE occurred in 33 patients (36%) in the DEB group, compared to 17 patients (19%) in the DES group (p log-rank = 0.02). After multivariate adjustment, there was no significant difference between the groups (HR for DEB = 1.45 [95%CI: 0.75-2.83]; p = 0.27). Mortality rates at 1 year were 11% with DEB, and 3% with DES (p = 0.04; adjusted HR = 2.85 [95%CI: 0.98-8.32]; p = 0.06).

**Conclusion:**

In a population with a high proportion of ACS, a non-significant numerical signal towards increased rates of MACE with DEB compared to second-generation DES for the treatment of ISR was observed, mainly driven by a higher mortality rate. An adequately-powered randomized controlled trial is necessary to confirm these findings.

## Introduction

Drug-eluting stents (DES) are considered as the standard of care in percutaneous coronary intervention across a broad range of lesion complexity,^1,2^ indications for revascularization,^3-6^ and patient categories.^7^ Treatment of in-stent restenosis (ISR) with DES improves outcomes compared to bare-metal stents (BMS) and balloon angioplasty.^8-10^ However, the long-term impact of using multiple metal layers in coronary arteries is not fully understood.^11^ Moreover, the use of DES requires long-term dual antiplatelet therapy (DAPT), significantly increasing bleeding risk, especially among patients requiring concomitant oral anticoagulation.^12^ Finally, despite low contemporary rates, stent thrombosis remains a catastrophic potential adverse event following DES implantation.^13,14^

Drug-eluting balloons (DEB) provide an alternative for revascularization that avoids the risk of thrombosis associated with stenting and reduces the risk of restenosis associated with standard balloon angioplasty and BMS. The use of a DEB for treatment of ISR has a robust cost-effectiveness profile as compared to DES over a one-year period, mainly owing to savings associated with DAPT.^15^ Prior studies have suggested that a stent-based drug-elution might not be necessary to prevent recurrent ISR.^16,17^ Yet, randomized trials comparing paclitaxel-eluting balloons to DES for ISR treatment have shown conflicting results with regards to angiographic endpoints.^18-23^ These studies have assessed clinical outcomes following DEB for ISR mostly as a secondary endpoint, enrolled mainly patients with stable coronary artery disease, and primarily used first-generation DES as the standard therapy for comparison. The objective of the present study was, therefore, to compare the clinical outcomes following DEB to second-generation DES for the treatment of ISR in a population comprised of a majority of patients presenting with an acute coronary syndrome (ACS).

## Methods

A retrospective cohort study was performed, comparing consecutive patients who underwent treatment of ISR using a paclitaxel-eluting balloon (Pantera Lux™, Biotronik, Berlin, Germany) to a random sample control group (1:1) of patients treated with a second-generation DES for ISR between December 2009 and November 2012, at the Centre Hospitalier de l'Université de Montréal (CHUM), an academic tertiary care centre (Canada). The selection of DEB or DES was left at the operator's discretion. Duration of DAPT was in accordance with the current practice guidelines for the specific indication for revascularization. DAPT was prescribed for a minimum of 3 months following elective DEB angioplasty. When performed, follow-up coronary angiography was clinically driven. Data were abstracted from electronic and paper medical records, and completed by telephone interviews. Coronary angiograms were independently reviewed by one investigator.

The primary outcome was a composite endpoint of major adverse cardiovascular events (MACE) including death from any cause, non-fatal myocardial infarction, and target lesion revascularization (TLR) at last follow-up. Secondary outcomes included device thrombosis, and the individual components of the primary outcome.

Endpoints were defined as per the Academic Research Consortium standardized definitions.^24^ The local institutional Ethics Committee approved the protocol in compliance with the Declaration of Helsinki, and a waiver of consent was obtained. The study was conducted according to the STROBE statement.^25^

### Statistical analyses

Continuous variables were presented as medians with 25-75% interquartile range (IQR). Categorical variables were expressed as proportions. Group comparisons of baseline characteristics were performed using the Pearson χ^2^ for categorical variables, and the Kruskal-Wallis test for continuous variables. Unadjusted comparison of the primary outcome between the DEB and DES groups was performed using the log rank test. One-year freedom from MACE and mortality were compared with the Pearson χ^2^ test. Freedom from MACE was illustrated using Kaplan-Meier curves. Multivariate Cox regression model was used to assess the impact of DEB on the primary and secondary outcomes. Covariates included in the multivariate model were based on a combination of a stepwise backward selection to identify independent risk factors for MACE in the cohort, and a priori knowledge of predictors of MACE (the latter variables being forced into the model). To limit over-fitting, the number of covariates retained was such that the ratio of events to covariates remained at least ten. From the available baseline and procedural characteristics, the stepwise selection process was used with an entry and stay criteria of 0.20 and 0.05, respectively. Interaction analyses were performed by adding an interaction term in the same multivariate Cox model to evaluate the relationship between DEB and MACE in the following pre-specified subgroups: DEB/DES length (≥ 20 mm or < 20 mm), diameter (≥ 3 mm or < 3 mm), and indication for revascularization (ACS or stable angina). In the DEB group, rates of MACE following treatment of intra-DES and intra-BMS restenosis were compared by using the same multivariate model as an exploratory analysis. Throughout the study, statistical significance was set at a two-sided p-value < 0.05. Statistical analyses were performed with SPSS^®^ Statistics 20.0 (IBM^®^, Armonk, NY).

## Results

From December 2009 to November 2012, DEBs were used in 100 patients, of whom 91 (91%) had follow-up data and were included in the analysis. The DES group included 89 patients treated with 6 zotarolimus-eluting stents (5 Endeavor^®^ and 1 Resolute Integrity^®^, Medtronic Vascular, Santa Rosa, CA) and 94 everolimus-eluting stents (93 Xience V™, Abbott Vascular, Santa Clara, CA; 1 Promus Element™, Boston Scientific, Natick, MA). Median follow-up was 24 months (IQR: 15 to 32 months) in the DEB group and 27 months (IQR: 20 to 33 months) in the DES group. Baseline clinical characteristics for both groups are presented in Table 1. ACS was the indication for revascularization in 65 patients (71%) in the DEB group and 69 patients (78%) in the DES group (p = 0.35) (total cohort: 134 patients [74%]). Procedural data are shown in Table 2. There were more focal lesions and fewer occlusive lesions in the DEB group compared with the DES group (p = 0.05). Intra-DES reevascularization (compared to intra-BMS revascularizaton) was more frequent in the DEB group (p = 0.01). Preparation of the lesion with a cutting balloon was more frequent in the DEB group (19% versus 2%; p < 0.001), and maximal inflation pressure was higher (median: 16 atm versus 14 atm; p = 0.03) in the DEB group.

**Table 1:** Baseline characteristics

	Drug-eluting balloon (n = 91)	Drug-eluting stent (n = 89)	p-value
Age (years)	66 (59-71)	66 (56-74)	0.89
Women	21 (23%)	24 (27%)	0.55
Body mass index (kg/m^2^)	28 (26-34)	27 (24-30)	0.01
Diabetes	43 (47%)	33 (39%)	0.29
Hypertension	80 (89%)	72 (84%)	0.32
Dyslipidemia	86 (97%)	81 (93%)	0.29
Previous Stroke/TIA	11 (13%)	11 (13%)	0.95
Chronic kidney disease	22 (28%)	26 (33%)	0.46
Previous CABG	26 (29%)	17 (20%)	0.14
**Indication**			**0.37**
Stable angina	26 (29%)	20 (23%)	
Unstable angina	36 (40%)	37 (42%)	
NSTEMI	26 (29%)	24 (27%)	
STEMI	3 (3%)	8 (9%)	

TIA: transient ischemic attack; CABG: coronary artery bypass graft; NSTEMI: non-ST elevation myocardial infarction; STEMI: ST-elevation myocardial infarction.

**Table 2:** Procedural characteristics

	Drug-eluting balloon (n = 91)	Drug-eluting stent (n = 89)	p-value
**Access site**			**0.64**
Radial	55 (60%)	59 (67%)	
Femoral	34 (37%)	28 (32%)	
Radial + femoral	1 (1%)	0 (0%)	
Brachial	1 (1%)	1 (1%)	
**Coronary territory**			**0.31**
Left main	3 (3%)	4 (5%)	
Left anterior descending	28 (31%)	29 (33%)	
Circumflex	27 (30%)	16 (18%)	
Right coronary artery	33 (36%)	40 (45%)	
DES ISR	55 (66%)	28 (42%)	0.01
Intra-CABG ISR	10 (11%)	8 (9%)	0.66
**ISR pattern**			**0.01**
Focal	52 (61%)	40 (46%)	
Diffuse	26 (31%)	25 (29%)	
Proliferative	4 (5%)	4 (5%)	
Occlusive	3 (4%)	18 (21%)	
**Adjunctive procedures**			
Rotational atherectomy	0 (0%)	1 (1%)	0.33
Thrombectomy	3 (3%)	7 (8%)	0.18
Cutting balloon	17 (20%)	2 (2%)	< 0.01
Balloon/stent diameter (mm)	3.00 (3.00-3.50)	3.00 (2.75-3.50)	0.61
Balloon/stent length (mm)	20 (20-30)	28 (18-30)	< 0.01
Maximal inflation pressure (atm)	16 (12-19)	14 (12-16)	0.03

DES: drug-eluting stent; ISR: in-stent restenosis; CABG: coronary artery bypass graft.

**Figure 1:**
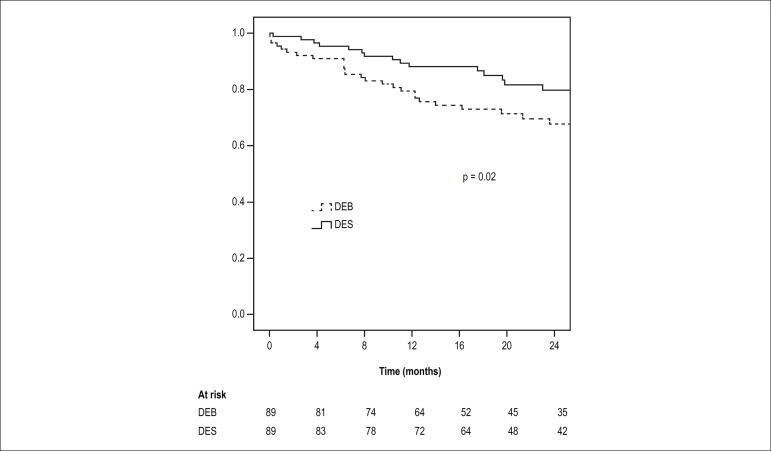
Unadjusted freedom from major adverse cardiovascular event. DEB: drug-eluting balloon; DES: drug-eluting stent.

**Figure 2:**
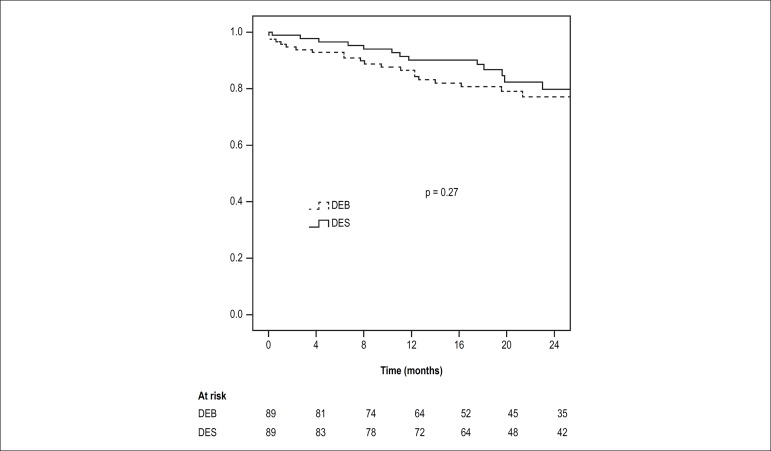
Adjusted Cox proportional hazards model analysis of major adverse cardiovascular event. DEB: drug-eluting balloon; DES: drug-eluting stent.

The primary outcome occurred in 33 patients (36%) in the DEB group, compared to 17 patients (19%) in the DES group (unadjusted p log-rank = 0.02). At one year, MACE occurred in 18 (23%) and 10 (12%) patients in the DEB and DES groups, respectively (Pearson χ^2^ p-value = 0.06). Freedom from MACE at follow-up is illustrated in Figure 1. Covariates included in the final multivariate model were age, body mass index, diabetes, chronic kidney disease stage ≥ 3a (defined as creatinine clearance < 60 mL/min according to the Cockroft-Gault formula), and ACS (versus stable angina) as the indication for revascularization. After multivariate adjustment, no significant difference in the rates of MACE between both groups was present (adjusted HR for DEB = 1.45 [95%CI: 0.75-2.83]; p = 0.27) (Figure 2). Secondary outcomes are shown in Table 3. Two in-hospital deaths occurred in each group. One-year mortality rates were 11% (10 patients) and 3% (3 patients), in the DEB and DES groups respectively (Pearson χ^2^ p-value = 0.04). Though numerically higher in the DEB group, all-cause mortality at follow-up (23% versus 7%) was not significantly different after multivariable adjustment (adjusted HR = 2.85; p = 0.06). One-year rates of TLR were 6% (5 patients) and 5% (4 patients), respectively (Pearson χ^2^ p-value = 0.75). In the DEB group, there was no significant difference between BMS-ISR and DES-ISR (adjusted HR = 0.90 [95%CI: 0.37-2.20] p=0.82) in terms of MACE.

**Table 3:** Primary and secondary outcomes following treatment of in-stent restenosis

	Drug-eluting balloon (n = 91)	Drug-eluting stent (n = 89)	Adjusted hazards ratio* (95% confidence interval)	p-value
MACE	36%	19%	1.45 (0.75-2.83)	0.27
All-cause death	23%	7%	2.85 (0.98-8.32)	0.06
Non-fatal myocardial infarction	9%	6%	1.40 (0.43-4.6)	0.58
Target-lesion revascularization	10%	8%	1.29 (0.44-3.76)	0.64
Binary restenosis	13%	9%	1.03 (0.37-2.88)	0.95
Lesion thrombosis	1%	0%	78.96 (N/A)	0.67
All-cause revascularization	24%	16%	1.23 (0.57-2.63)	0.60

MACE: major adverse cardiovascular events.

* Adjusted Cox proportional hazards model, including age, body mass index, diabetes, chronic renal disease, and acute coronary syndrome as the indication for revascularization.

No modification of the effect of DEB on the occurrence of MACE was observed for balloon-stent diameter (3 mm versus ≥ 3 mm) (p for interaction = 0.92), balloon/stent length (< 20 mm versus ≥ 20 mm) (p for interaction = 0.77) or ACS as the indication for revascularisation (p for interaction = 0.45).

## Discussion

In the present study, assessing long-term clinical outcomes in a real-world primarily ACS population, we found that ISR treated with a paclitaxel-eluting balloon, compared to second-generation DES, while not showing a significant difference in overall MACE rates after adjustment, might be associated with a higher all-cause mortality rate. The present study was designed as an exploratory analysis of a real-world population and it can neither prove the clinical superiority or non-inferiority of DEB compared to DES for ISR. Rather, given the paucity of data on the clinical outcomes of DEB compared with current standard practice for this indication, we sought to add to the literature providing comparative clinical data on the use of DEB and second-generation DES in a real-world setting. Strengths of the analysis include an all-comer cohort presenting mostly with ACS, and use of second-generation DES as a comparator, both reflecting more accurately current clinical practice than previous reports.^18,19,21^ Though relatively small, the sample size was similar to previous clinical trials of DEB.^18,19,21-23^ The results of the present study are relevant for patient care optimization as concerns remain regarding DES for treatment of ISR despite their proven short-term efficacy.

The angiographic efficacy of DEB compared to first-generation DES has previously been demonstrated.^18-21^ However, the RIBS IV randomized trial showed that everolimus-eluting stents were associated with improved angiographic outcomes compared to the SeQuent® Please DEB for treatment of DES-ISR.^22^ Clinical events in DEB-ISR trials were only reported as secondary endpoints. In addition, only a minority of patients presented with an ACS in these trials, and none enrolled patients with an acute myocardial infarction. In the ISAR-DESIRE-3 trial, rates of MACE (23.5%) in the DEB group at one year were comparable to the rates in our cohort (23%).^20^ In the present study, the mortality rate in the DEB group at one year (11%) was higher than in ISAR-DESIRE-3 (2.2%), suggesting that our real-world cohort might have represented a higher-risk population. This hypothesis is supported by the fourfold higher rate of ACS in our cohort (77%) compared to ISAR-DESIRE-3 (19%). In the PEPCAD-II trial, there was, in contrast to our findings, a strong trend towards lower rates of MACE in the DEB group compared to paclitaxel-eluting stent (9% versus 22%, respectively; p = 0.08).^21^ However, in addition to being compared to first-generation DES, there were only 5 total deaths in the PEPCAD-II trial, suggesting again a population at lower overall risk than the one in this study.^21^ Previous trials (except for RIBS IV) used first-generation DES as comparators, and this might at least in part explain why the signal observed in our study in disfavour of DEB was not observed previously.^18,19,21,23^ Patients with ACS might still benefit more from a second-generation DES over a DEB for treatment of ISR, as high local and systemic pro-thrombotic and inflammatory milieu of ACS might not be suitable for DEB use, but this hypothesis remains to be confirmed.

Limitations of the present analysis include its non-randomized, single-centre, retrospective design. Selection bias was likely, and while multivariate modelling appeared to adequately account for known confounders, unmeasured confounding might remain. Additionally, it was not adequately powered to detect differences in rare clinical endpoints, such as device thrombosis. However, the sample size in this study is on par with those from prior clinical trials of DEB for the treatment of ISR.^16-19,21,23^ Also, the current analysis lacks information on the duration of DAPT following ISR angioplasty. Future trials should address the efficacy of DEB in the setting of ACS and seek to define current clinical practice regarding DAPT following DEB, as the duration of DAPT and its associated costs and complications may prove to be the determining factors in the event of ongoing clinical equipoise between DEB and second-generation DES.

## Conclusion

In conclusion, the present study showed that in a population with a high proportion of ACS, a non-significant numerical increase in MACE was observed with the use of DEB to treat ISR compared to second-generation DES. It was mainly driven by a concerning trend toward higher mortality with the use of DEB. Confirmation of these results by an adequately-powered randomized trial in the ACS population with clinically-driven endpoints is paramount to appropriately clarify the role of DEB in the interventional cardiology armamentarium.
